# Does higher perceived risk of morbidity and mortality decrease risk-taking?

**DOI:** 10.1098/rsos.220486

**Published:** 2022-12-07

**Authors:** Mélusine Boon-Falleur, Brigitte Dormont, Coralie Chevallier

**Affiliations:** ^1^ LNC², Département d’études cognitives, Ecole normale supérieure, Université PSL, INSERM, 75005 Paris, France; ^2^ LEDa, Université Paris-Dauphine, Université PSL, IRD, CNRS, 75016 Paris, France

**Keywords:** risk-taking, patience, perceived risk, structural model, COVID-19

## Abstract

Previous studies have shown that people change their behaviour in response to negative shocks such as economic downturns or natural catastrophes. Indeed, the optimal behaviour in terms of inclusive fitness often varies according to a number of parameters, such as the level of mortality risk in the environment. Beyond unprecedented restrictions in everyday life, the COVID-19 pandemic has profoundly affected people's environment. In this study, we investigated how people form their perception of morbidity and mortality risk associated with COVID-19 and how this perception in turn affects psychological traits, such as risk-taking and patience. We analysed data from a large survey conducted during the first wave in France on 3353 nationally representative people. We found that people use public information on COVID-19 deaths in the area where they live to form their perceived morbidity and mortality risk. Using a structural model approach to lift endogeneity concerns, we found that higher perceived morbidity and mortality risk increases risk aversion. We also found that higher perceived morbidity and mortality risk leads to less patience, although this was only observed for high levels of perceived risk. Our results suggest that people adapt their behaviour to anticipated negative health shocks, namely the risk of becoming sick or dying of COVID-19.

## 1. Introduction

Behavioural ecology has a long tradition of studying how organisms—whether humans or other species—adapt to different parameters in their environment [1]. In particular, life-history theory studies how organisms allocate resources between growth, maintenance and reproductive functions and how this allocation depends on parameters such as age, sex, mortality risk or access to resources [2]. Optimal allocation of resources in terms of maximizing inclusive fitness depends on a number of trade-offs. For example, an individual may have to decide between investing resources in maintenance to increase their lifespan or investing resources in finding a mate to increase their chances of reproduction [3]. Originally applied to differences across species, the life-history framework is now also applied to study within species differences based on environmental factors [4,5]. Looking at inter-individual differences, life-history theory states that the optimal decision depends, among other parameters, on whether the individual is in a harsh environment with high mortality risk or in a favourable environment with a lower mortality risk [6]. For individuals living in harsh environments, it makes more sense to focus on reproductive efforts than on long-term health and somatic maintenance [7]. Similarly, the behavioural ecology literature studies how animals adapt their behaviour to their environment and makes a number of predictions related to the prevailing mortality risk [8].

Both theoretical and empirical research has indeed shown that people calibrate their behaviour in response to environmental adversity [9–13]. For example, Lee *et al*. found that individuals in countries with low life expectancy were more likely to prefer an immediate reward to one that is delayed, suggesting that cues to mortality can affect how individuals make decisions about the future [14]. Among the various behavioural responses to adversity, time discounting and risk-taking are often studied, as they inform a number of life-history trade-offs. When facing increased mortality risk, models show that individuals should discount the future more, both because of collection risks [12,14,15] and waiting costs [16,17]. Regarding risk-taking, a dominant view in the literature argues that being risk-seeking is adaptive when facing adverse conditions, because individuals may be unlikely to achieve their goal with a safe strategy and may instead decide to maximize the probability of achieving their goal by adopting a riskier strategy [10,11,18]. However, this prediction relies on a definition of risk-taking that is not based on the variance of outcomes, but on the possible cost of outcomes (hazardous risk) [19]. Instead, if one considers risk-taking as the variance in pay-offs that an individual is willing to accept, then the prediction is reversed: individuals facing adverse conditions should take fewer risks as they want to minimize potential losses [20,21].

The effect of negative shocks on time discounting and risk-taking have been studied in numerous contexts such as natural disasters [22–25], conflicts or violence trauma [26,27] and financial adversity [28–30]. For example, early-life financial experiences such as the Great Depression are linked to more conservative financial investments later in life, suggesting less risk-taking [30]. People who experience close bereavement also have steeper time discounting [31], and higher extrinsic mortality perception is associated with diminished investment in preventative health [32–34]. Being exposed to higher risk of sickness or death also has an impact on people's preferences and behaviour. Studies have shown that a negative health shock decreases people's risk-taking [35], suggesting that people prefer to ‘play it safe’ when the threat of illness is high. One important distinction is whether the mortality threat is extrinsic or intrinsic in nature. In contexts where the mortality threat is more uncontrollable, individuals adopt more present-oriented preferences and engage in more hazardous behaviours [7,36–38]. In contexts where threats are more controllable, such as when social distancing can mitigate the risk, we expect individuals to engage in fewer hazardous behaviours and their time preferences to be less affected [18,35,39], with the exception of people who feel especially vulnerable to those threats [38].

In this pre-registered study, we investigated the impact of an increased morbidity and mortality risk linked to the COVID-19 pandemic. We conceptualize people's adaptation to this negative health shock as a two-step process. First, people assess their own likelihood of becoming sick or dying because of COVID-19 based on the information that is available to them. Second, based on their assessment, people adapt their behaviour in ways that maximize their fitness. In this study, we are interested in measuring risk preference in terms of the variance of outcomes rather than risk in terms of hazardous behaviours in specific domains (e.g. ignoring health guidelines). Engaging in hazardous behaviours may be caused by many factors, such as the perceived cost of these behaviours, and thus may not perfectly capture the underlying preference for risk. Therefore, we focus on economic risk, which only captures preferences in terms of variance in outcomes.

We first studied the factors that influenced people's perceived risk of becoming sick or dying because of COVID-19. Research has shown that the assessment of risk is influenced by emotional, cognitive, social and cultural variations both between individuals and between countries [40–46]. In addition, people's risk perception is influenced by the information that is available to them, both publicly and privately. For example, people who spent more time on social media perceived higher mortality and morbidity risk associated with COVID-19, probably because of the frequency of negative feelings expressed in such media [47,48]. The pandemic thus provides a unique opportunity to study how people form their expectations about being infected or suffering from other adverse health consequences. We expect that people take into account all relevant information in their environment, including their own exposure to the disease through their occupation, or through the local prevalence of the virus. We then explored how people perceived morbidity and mortality risk in turn influences their behaviour. In particular, we hypothesized that people perceiving a high morbidity or mortality risk would become less patient in terms of economic discounting, and would take less risk in terms of economic variance.

To test these hypotheses, we collected data at the end of the first wave of the COVID-19 pandemic in France (June 2020). Participants in our study came from all over the country, thus allowing us to exploit regional variation in the incidence of COVID-19 cases to study how the prevalence of the virus affected perceived morbidity and mortality risk, and how this perception in turn affected patience and risk-taking. This unique dataset allowed us to lift a pervasive endogeneity concern in the literature. Indeed, baseline patience or risk aversion might well have an effect on people's experience of the COVID-19 health shock. For example, people who are more risk-seeking might have a more unhealthy lifestyle or might expose themselves more to diseases, and might therefore be more likely to experience a health shock. This would lead to a positive correlation between perceived morbidity and mortality risk on the one hand and risk-taking on the other hand. In addition, other unobserved confounding factors such as neuroticism may influence both perceived morbidity and mortality risk, and patience and risk-taking. If this were true, standard regressions would yield a biased estimate of the relationship between perceived morbidity and mortality risk and patience (or risk-taking).

To address these endogeneity concerns, we used a structural model. This econometric method allowed us to compute an estimate that is solely based on the fraction of perceived morbidity and mortality risk variability that is correlated to an exogenous variable, named the instrumental variable. For the purpose of this study, we used the percentage of COVID-19-related deaths in the geographic area as an instrument for perceived morbidity and mortality risk. There were a total of 96 different areas in the study, based on the administrative area in which people lived. In the case of the COVID-19 pandemic, our rationale was that this public information on COVID-19 morbidity at the local level was exogenous to people's psychological traits and thus constitutes a valid instrument [49]. This approach allowed us to study the causal link of an anticipated shock (the expectation of becoming sick or dying from COVID-19 in the future) on behaviour.

## 2. Methods

### 2.1. Ethics information

The study design and data collection were conducted according to the ICC–ESOMAR international code for market research, social and opinion studies and data analytics. In addition, data collection, storage and analysis respected applicable data protection laws and all the new rules related to the European GDPR regulations.^1^ All participants had to give their informed consent to be surveyed. The data were made publicly available after being made anonymous. The geographical information included in our study is coarse enough to prevent indirect identification of individual participants.

### 2.2. Sampling plan

Our data come from one module of a weekly repeated survey conducted by DataCovid from 7 April to 9 June 2020 that tracked individuals’ reactions and behaviours in France during the pandemic. The data were collected online by the polling company Ipsos and participants received a small compensation for their participation in the survey. The weekly survey was conducted on a different sample for each wave such that we do not have longitudinal data on people's attitudes. The data collection corresponds to the 8th wave of this survey. It took place at the beginning of June 2020, a few weeks after the end of the first lockdown imposed by the French government (the first French lockdown took place between 16 March and 11 May 2020). In line with the needs of our empirical evaluation, the period in which the data were collected coincides with a period in which regional disparities in the prevalence of COVID-19 were important, and in which all citizens were still subjected to the same regulations (such as maximum capacities in bars and restaurants). As a result, two individuals sampled randomly across the French territory experienced similar lifestyle changes, while being exposed to different numbers of COVID-19 cases and deaths. A sample of 5000 French adult citizens was surveyed. The sampling process was stratified to ensure representativeness in terms of age, sex, socioeconomic status, area of residence and population density of the place of residence (e.g. urban versus suburban). We excluded participants who responded ‘I don't know’ to one or more questions about perceived risk and respondents who responded ‘I prefer not to say’ for the question about their income, leading to a final sample size of *N* = 3353. A summary of participants' personal characteristics can be found in table 1.

**Table 1.** Summary statistics. For each participant, we recorded several demographic variables: gender, age group, whether the individual was a health worker (health worker), whether the individual had to work outside their home during the lockdown (working outside their home), whether a family member or close friend was sick or died as a result of COVID-19 (family or friend had COVID-19), the individual's perceived combined morbidity and mortality risk (low perceived risk (1%), medium perceived risk (between 1% and 25%) and high perceived risk (25%)). The total number of participants included in the sample was *N* = 3353.

statistic	number of individuals	percentage of total sample (%)
female	1773	52.9
age (18–24)	247	7.4
age (25–34)	560	16.7
age (35–44)	640	19.1
age (45–54)	616	18.4
age (55–64)	568	16.9
age (> 65)	722	21.5
health worker	33	1.0
working outside their home	1080	32.2
family or friend had COVID-19	366	10.9
low perceived risk	931	27.8
medium perceived risk	1856	55.3
high perceived risk	566	16.9

### 2.3. Design

Each participant completed the same online survey for a total duration of approximately 16 min. The first part of the survey consisted of demographic questions (age group, gender, area of residence and socioeconomic status). The second part of the survey included COVID-19-related questions, such as respondents' current work situation (e.g. furloughed, working from home, unemployed, etc.). The third part of the survey included five questions measuring respondents' risk-taking and five questions measuring their patience. The final part of the survey assessed respondents' perception of their COVID-19 mortality and morbidity risk. All data were analysed using the software STATA. In the following paragraphs, we provide details of the different measures used in the survey.

#### 2.3.1. Measure of perceived morbidity and mortality risk

**Figure 1.**
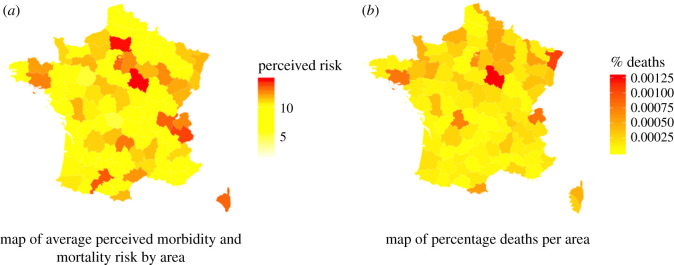
Map of COVID-19 deaths and perceived risk. (*a*) The combined perceived risk of being infected, getting very sick or dying from COVID-19 in early June 2020. The data come from the DataCovid Project, a citizen science initiative. (*b*) The percentage of deaths from COVID-19 in the total population by area in early June 2020. Data come from the French government's official statistics.

Our measures of perceived morbidity and mortality risk consisted of the following questions: (i) What is the probability that you become infected with SARS-CoV-2? (ii) What is the probability that you become very sick as a result of being infected with SARS-CoV-2? (iii) What is the probability that you die as a result of contracting COVID-19? For each question, respondents answered on a scale with six values 1%, 5%, 10%, 15%, 20% and 25%. These cut-off points were deemed to reflect the reasonable actual range of risk at the time of the data collection, although the perceived risk may have been higher. For each question, participants also had the possibility of answering ‘I don't know’. We looked at the three measures independently and also created a combined index of risk perception by computing the average of the three risk probabilities. Figure 1*a* shows that there are large geographical disparities in the average level of combined morbidity and mortality risk perception. For example, individuals living in Ile-de-France, the region that encompasses Paris, perceived that they were exposed to higher morbidity and mortality risk compared with individuals in the Southwestern part of France.

Looking at the distribution of perceived risk, we found that it followed a bimodal distribution, as many individuals reported 1% perceived risk, many people reported 25% risk, and the rest was uniformly distributed in between (see appendix, figure 3 for the histogram of risk perception). We, therefore, combined these categories to create a categorical variable with three values: low perceived risk (1%), medium perceived risk (between 1% and 25%) and high perceived risk (25%). The distribution of risk perception was the following: 28% of participants reported low perceived risk, 55% of participants reported medium perceived risk and 17% of participants reported high perceived risk.

#### 2.3.2. Measure of patience and risk-taking

Each participant completed the time and risk preferences tasks developed by Falk *et al*. [50]. For the measure of patience, each participant had to choose between a sure payment today and a varying sure payment in 12 months in five different situations (e.g. Would you rather receive 100€ today or 150€ in 12 months?). The proposed amount for which the individual prefers the payment in 12 months over the payment today was then used to infer the individual's patience. Similarly for risk-taking, participants had to choose between a lottery and a varying safe option in five different situations (e.g. What would you prefer: a 50 per cent chance of winning 300€ when at the same time there is 50 per cent chance of winning nothing, or would you rather have the amount of 160€ as a sure payment?). The proposed amount for which the individual prefers the safe option to the lottery was then used to infer risk-taking. More details about the distribution of these variables can be found in the appendix.

#### 2.3.3. Measure of COVID-19 geographical prevalence

We collected data from the French health authorities in order to compute the cumulative number of individuals who (i) were infected with SARS-CoV-2, (ii) were admitted in an intensive care unit as a result of COVID-19, and (iii) died as a result of COVID-19 in different areas since the beginning of the pandemic to the time of our study. These numbers were publicly released by the health authorities on a regular basis. To measure risk exposure, we computed frequencies by dividing each of these numbers by the total population of each area. Figure 1*b* displays the percentage of deaths from COVID-19 in each area. As expected, there was a lot of variation between areas during the first wave of the COVID-19 pandemic, with the Eastern and Parisian regions being most affected. Because the percentage deaths by area was not normally distributed, we took the natural log of percentage deaths to obtain a normally distributed variable.

#### 2.3.4. Additional covariates

We expect that individuals form their perception of morbidity and mortality risk related to COVID-19 based on different sources of information, e.g. personal health characteristics (co-morbidities, age, pregnancy, etc.), personal exposure to the virus (occupation, public transportation use, etc.) and experience with the disease (knowing someone who was sick or died as a result of being infected with SARS-CoV-2). For each participant, we recorded a number of demographic variables (age, gender, income, job status, educational attainment). Age was recorded as a categorical variable taking six values (18–24, 25–34, 35–44, 45–54, 55–64 and 65 years or more). Income was recorded as a categorical value, from 1 = less than 500€ per month, to 7 = more than 3000€ per month. A total of 511 participants selected ‘I prefer not to say’ for the question on income. We excluded these participants from our analysis. Educational attainment was also recorded as a categorical value, from 1 = no diploma, to 6 = three or more years of higher education. A total of eight participants did not report their educational attainment and were excluded from our study. Each participant had to state whether they were a health worker and whether they worked outside their home during the lockdown (as opposed to individuals who were either put on furlough or remote workers). See table 1 for more details.

### 2.4. Statistical analysis

The first question we want to address is how do people assess their morbidity and mortality risk, given the information available to them. We expect that people use both individual-level data, such as their age, sex and occupation, and group-level data, such as COVID-19 prevalence where they live. We first looked into the factors that influence people's perceived morbidity and mortality risk by running an ordered probit model. We regressed the perceived morbidity and mortality risk (low, medium or high) on gender, age group, income group, educational attainment, being a health worker, working outside their home (as opposed to being furloughed, unemployed or working from home), and the log of percentage COVID-19-related deaths in an individual's area. We are particularly interested in the effect of the percentage of COVID-19-related deaths in an individual's area on that individual's perceived morbidity and mortality risk. We expect that higher COVID-19 death prevalence will lead to higher perceived risk.

The second part of our analysis investigated the impact of perceived morbidity and mortality risk on patience and risk-taking. Such analysis potentially runs into the problem of reverse causality and endogeneity. Indeed, perceived morbidity and mortality risk could affect risk-taking and patience, but the converse may also be true. In addition, other psychological factors, such as anxiety, may be causing both perceived morbidity and mortality risk and patience or risk-taking to increase. As a result, using a standard regression of risk-taking and patience on perceived mortality and morbidity risk does not allow us to draw any causal inference and may yield biased estimates. To address this issue, we implemented a structural modelling approach. To do so, we must first identify an exogenous variable that affects perceived morbidity and mortality risk, without affecting other psychological traits. Such a variable is used as an ‘instrument’ to capture the exogenous variation in perceived morbidity and mortality risk (equation (2.1)). We chose the publicly available percentage of deaths by department as an instrument. Our rationale was that in the early days of the pandemic, the spread of COVID-19 was mostly due to random events (such as a superspreading event in the eastern part of France), and thus was likely to be uncorrelated to people's psychological traits. As a result, the variation in perceived morbidity and mortality risk that is correlated to variations in the percentage of deaths in an area is likely to be unrelated to psychological factors. Once this exogenous variation in perceived morbidity and mortality risk is identified, it can be used to estimate the impact of perceived morbidity and mortality risk on risk-taking and patience (equation (2.2)).

2.1 $$x_{i}=aZ_{d}+bW_{i}\;+\mu_{i}$$

and

2.2 $$y_{\;j,i}\;=\beta_{j}x_{i}+\delta_{j}W_{i}\;+\varepsilon_{\;j,i},$$

where *x_i_* is the subjective morbidity and mortality risk perception, *Z*_d_ is the percentage of COVID-19-related deaths in the area, *W_i_* is a vector of control variables, measured at the individual level (age, gender, income), and denoting *y_1,i_* the individual's time discounting factor and *y_2,i_* the individual's coefficient of risk aversion. In this model the non-exogeneity of perceived risk takes the form of a correlation (*ρ*) between the disturbances of equations (2.1) and (2.2). Estimating this model allows us to obtain consistent estimates even if perceived risk is non-exogenous and to test its exogeneity through the test *ρ* = 0.

Given that subjective risk is a categorical variable (with low, medium and high values), the first equation is specified as an ordered probit model estimated by the maximum-likelihood estimator. The second equation is a simple linear model. To deal with the possible correlation between the disturbances of equations (2.1) and (2.2), we estimate our bivariate model with a maximum-likelihood estimator, where the likelihood is written for the two equations at once. This approach leads to a consistent (unbiased) and efficient estimation. However, if the exogeneity of the subjective risk is not rejected, it is possible to use it directly in equation (2.2) without using the instrument provided by public information on risk. In this case it is preferable to use a semi-parametric approach such as ordinary least squares, as it is less constraining than the maximum likelihood, which is based on an assumption on the data-generating process law.

## 3. Results

### 3.1. Morbidity and mortality risk perception

**Figure 2.**
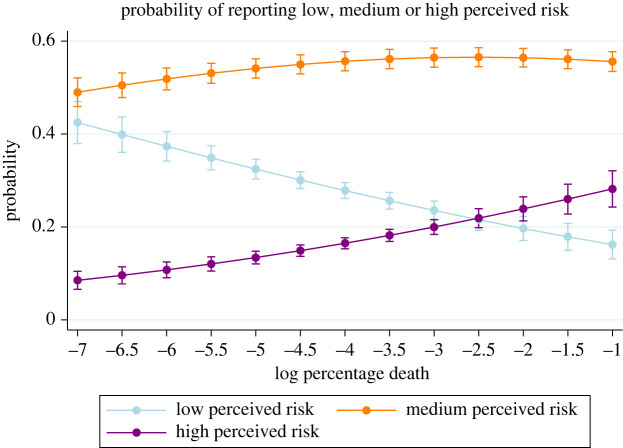
Probability of reporting low, medium or high perceived morbidity and mortality risk depending on the log percentage of COVID-19-related deaths in the area. We found that the percentage of deaths had a negative marginal effect on the probability of perceiving a low risk, and a positive marginal effect on the probability of perceiving a high risk. There is no clear marginal effect on the probability of perceiving a medium risk. The bars represent the 95% confidence interval. *N* = 3353. We excluded participants who responded ‘I don't know’ to one or more questions about perceived risk and respondents who responded ‘I prefer not to say’ for the question about their income.

**Figure 3.**
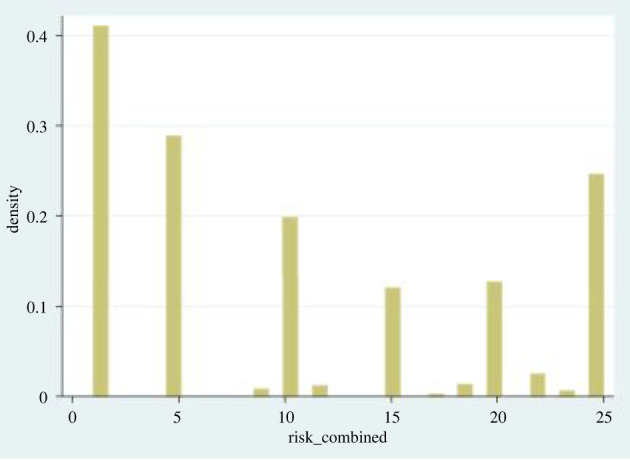
Histogram of the combined perceived risk (*N* = 3642, we excluded participants who answered ‘I don't know’ to one or more questions about perceived risk).

We found that women perceived higher risk than men, although men were at higher risk of dying than women (*β* = 0.084 and *p* < 0.05, table 2 for details). This is consistent with other studies of perceived risk associated with COVID-19, and it may be due to differences in occupational exposure [51,52]. We also found that older individuals perceived lower risk than younger individuals (table 2). This could be explained by the fact that older people, although they have higher morbidity and mortality risk if they become infected with SARS-CoV-2, were also less exposed to the virus because of social distancing. These results are similar to other studies related to perceived risk during the COVID-19 pandemic. We also found that people's occupation was correlated with their risk perception: individuals working in the health sector and individuals who worked outside their home (as opposed to working remotely, being unemployed, or being on furlough) reported higher perceived morbidity and mortality risk (*β* = 0.799 and *p* < 0.000 and *β* = 0.307, *p* < 0.000, respectively, table 2). Finally, we found that, as predicted, the probability of reporting low perceived risk decreased with the percentage of COVID-19-related deaths in the area, while the probability of reporting high perceived death increased (figure 2). Given that educational attainment, especially in terms of numerical ability, has been shown to strongly influence perceptual biases and participant responses to risk-related questions [53], we included dummies for educational attainment in our model and found no effect (see appendix, table 6).

**Table 2.** Perceived risk as an ordered probit model. This table shows an ordered probit model for perceived risk as a function of the log of percentage deaths in the individual's area, whether that person worked outside their home during the first confinement, whether that person was a health worker, and a number of demographic variables. The reference age group is 35–44 years old, and the reference income group is 1000–1500 euros per month. *N* = 3353. We excluded participants who responded ‘I don't know’ to one or more questions about perceived risk and respondents who responded ‘I prefer not to say’ for the question about their income.

predictor variable	coefficient	s.e.
percentage death	0.136***	0.019
work outside their home	0.307***	0.051
health worker	0.799***	0.227
sex (female)	0.084*	0.041
age 18–24	0.070	0.080
age 25–34	0.027	0.080
age 45–54	–0.203**	0.072
age 55–64	–0.327***	0.072
age 65 and over	–0.135*	0.067
income (less than 500 euros)	–0.118	0.128
income (500–800 euros)	–0.107	0.112
income (800–1000 euros)	–0.038	0.113
income (1500–2000 euros)	0.058	0.078
income (2000–3000 euros)	0.104	0.066
income (more than 3000 euros)	0.123*	0.060

* *p* < 0.05, ** *p* < 0.01, *** *p* < 0.001.

### 3.2. The effect of perceived risk on risk-taking and patience

We then tested whether perceived morbidity and mortality risk affected risk-taking and patience. We first checked whether our measures of risk-taking and patience were consistent with the literature. We regressed risk-taking and patience on gender, age and income. Similarly to Falk *et al.* [54], we found that women take fewer risks and are less patient, and that risk-taking decreases with age (table 3). However, we did not find a positive correlation between age and patience unlike the results obtained in the studies conducted by Falk *et al.* [50]. Consistent with other results in the literature, we found that higher income groups were associated with higher risk-taking and patience (table 3 for results) [19]. Based on prior research, we expected that individuals who were at risk of losing their job or of having their business go bankrupt would be less risk-taking and less patient [30]. We regressed risk-taking and patience on the probability of losing one's job or going bankrupt while controlling for gender, age and income (see appendix, table 6). In line with our hypothesis, we found that people who had a higher chance of losing their job were less risk-taking, although not significantly so (*β* = –0.001, *p* = 0.142), and significantly less patient (*β* = –0.004, *p* < 0.000). These analyses collectively suggest that our survey adequately measured risk-taking and patience.

**Table 3.** Regression of risk-taking and patience on demographic variables. Method: linear regression of risk-taking and patience on a number of control variables. The reference age group is 35–44 years old, and the reference income group is 1000–1500 euros per month. *N* = 4489. We excluded participants who responded ‘I prefer not to say’ for the question about their income.

predictor variable	risk-taking		patience	
	*b*	s.e.	*b*	s.e.
sex (female)	–0.131***	0.027	–0.187***	0.049
age 18–24	0.298***	0.070	0.191	0.107
age 25–34	0.112**	0.037	0.166**	0.062
age 45–54	–0.010	0.041	–0.015	0.063
age 55–64	–0.117**	0.041	–0.038	0.063
age 65 and over	–0.142**	0.042	–0.112+	0.058
income (less than 500 euros)	–0.059	0.136	–0.275	0.167
income (500–800 euros)	–0.187+	0.096	–0.189	0.135
income (800–1000 euros)	–0.099	0.092	–0.166	0.120
income (1500––2000 euros)	0.069	0.055	0.162	0.082
income (2000–3000 euros)	0.147*	0.060	0.242***	0.070
income (more than 3000 euros)	0.303***	0.055	0.528***	0.067

* *p* < 0.05, ** *p* < 0.01, *** *p* < 0.001.

We then regressed risk-taking and patience on perceived morbidity and mortality risk using the structural modelling approach. As expected, we found that higher perceived morbidity and mortality risk was associated with less risk-taking (*β* = –1.17, *p* < 0.000, table 4). In addition, the coefficient *ρ* was significantly different from zero (*ρ* = 0.76, *p* < 0.000), which shows that our structural model is consistent. To check the robustness of our results, we ran a similar model using a different specification for perceived risk and found similar results (see appendix, tables 8 and 9).

**Table 4.** Estimation of a two-equation model explaining risk-taking and patience as a function of perceived risk. Method: maximum-likelihood estimator for the two-equation model, equation (2.1) explaining perceived risk as an ordered probit of the log of percentage of deaths in the area (bottom part of the table) and equation (2.2) explaining risk-taking and patience as a function of perceived risk (top part of the table). The reference age group is 35–44 years old, and the reference income group is 1000–1500 euros per month. *N* = 3353. We excluded participants who responded ‘I don't know’ to one or more questions about perceived risk and respondents who responded ‘I prefer not to say’ to the question about their income. We see that *ρ* (the correlation coefficient between the disturbances of equations (2.1) and (2.2)) is significantly positive for risk-taking, meaning that perceived risk is not exogenous. We see that *ρ* is not significantly different from zero, meaning that perceived risk is exogenous for patience. So, we must prefer a simple regression model. Indeed, in the case of exogeneity of the subjective risk, ordinary least squares are consistent like the maximum-likelihood estimation but more efficient.

predictor variable	risk-taking		patience	
	*b*	s.e.	*b*	s.e.
equation (2.2)				
perceived risk	–1.166***	0.112	0.079	0.410
sex (female)	–0.075+	0.039	–0.163**	0.061
age 18–24	0.300**	0.097	0.238	0.127
age 25–34	0.159*	0.074	0.201**	0.070
age 45–54	–0.156*	0.072	0.005	0.084
age 55–64	–0.363***	0.076	0.019	0.124
age 65 and over	–0.312***	0.059	–0.078	0.108
income (less than 500 euros)	–0.241	0.186	–0.225	0.191
income (500–800 euros)	–0.241+	0.126	–0.089	0.153
income (800–1000 euros)	–0.081	0.140	–0.131	0.163
income (1500–2000 euros)	0.147	0.083	0.154	0.103
income (2000–3000 euros)	0.202*	0.083	0.287**	0.094
income (more than 3000 euros)	0.390***	0.080	0.529***	0.093
equation (2.1)				
perceived risk	0.086***	0.016	0.131***	0.019
sex (female)	0.073	0.040	0.069	0.040
age 18–24	0.002	0.083	–0.014	0.081
age 25–34	0.024	0.080	0.019	0.080
age 45–54	–0.201**	0.074	–0.207**	0.073
age 55–64	–0.410***	0.071	–0.420***	0.071
age 65 and over	–0.301***	0.062	–0.304***	0.061
income (less than 500 euros)	–0.194	0.140	–0.186	0.133
income (500–800 euros)	–0.157	0.117	–0.158	0.112
income (800–1000 euros)	–0.098	0.112	–0.090	0.112
income (1000–1500 euros)	0.085	0.080	0.086	0.080
income (2000–3000 euros)	0.124	0.065	0.121	0.066
income (more than 3000 euros)	0.154*	0.061	0.150*	0.061
*ρ*	0.755***	0.037	−0.008	0.226

* *p* < 0.05, ** *p* < 0.01, *** *p* < 0.001.

For patience, however, we found no significant effect of perceived morbidity and mortality risk (*β* = 0.079, *p* > 0.05, table 4). The coefficient *ρ* was not significantly different from zero (*ρ* = –0.008, *p* > 0.05), which means that perceived risk is exogenous and the structural model is not efficient. We, therefore, ran a simple regression of patience on perceived risk, treating perceived risk as a set of dummies to impose the fewest constraints on the model. We found that the relationship between perceived risk and patience was not consistent: higher perceived risk was associated with less patience when going from a medium perceived risk to a high perceived risk (*β* = –0.222, *p* < 0.000) but lower perceived risk was associated with less patience as compared with medium perceived risk (*β* = –0.144, *p* < 0.05), see table 5.

**Table 5.** Patience as a function of perceived risk. Method: ordinary least squares. We regress patience on two dummies for perceived risk, one dummy for low perceived risk, and one dummy for high perceived risk. *N* = 3353. We excluded participants who responded ‘I don't know’ to one or more questions about perceived risk and respondents who responded ‘I prefer not to say’ for the question about their income. We find an inconclusive result: both lower and higher perceived risk seem to lead to lower patience.

predictor variable	patience	
	*b*	s.e.
perceived risk = low	–0.222***	0.059
perceived risk = high	–0.144*	0.059
sex (female)	–0.156**	0.058
age 18–24	0.207	0.126
age 25–34	0.202**	0.068
age 45–54	0.002	0.068
age 55–64	0.012	0.078
age 65 and over	–0.086	0.070
income (less than 500 euros)	–0.205	0.191
income (500–800 euros)	–0.085	0.158
income (800–1000 euros)	–0.137	0.162
income (1500–2000 euros)	0.156	0.098
income (2000–3000 euros)	0.280**	0.086
income (more than 3000 euros)	0.522***	0.077

* *p* < 0.05, ** *p* < 0.01, *** *p* < 0.001.

## 4. Discussion and limitations

We found that morbidity and mortality risk perception depends not only on personal characteristics such as age, gender and occupation, but is also affected by the actual prevalence of morbidity and mortality in people's area. Such information was publicly available and broadcast in many news outlets. This finding suggests that people were sensitive to the public news on the geographical prevalence of the pandemic, i.e. that they used the available information on risk to form their own beliefs.

Our structural model approach allowed us to identify a causal effect of perceived morbidity and mortality risk on psychological variables. We found that, as expected, higher morbidity and mortality risk perception was associated with less risk-taking. In line with recent work in behavioural ecology, our interpretation is that individuals want to minimize the chance of a loss in adverse contexts, and thus prefer the safer option [55]. People may want to minimize the economic risk they expose themselves to when they risk being sick, either directly because of the negative health shock, or indirectly because they expect to lose resources when they experience a negative health shock. For example, people who perceive a high morbidity and mortality risk could also perceive a higher chance of having to spend a lot of money on healthcare, a higher chance of being fired, or losing income due to inability to work for freelance workers. Turning to patience, contrary to the predictions made in the behavioural ecology literature, we did not find conclusive results for an effect of perceived risk on patience.

The fact that perceived morbidity and mortality risk is endogenous in the case of risk-taking and exogenous in the case of patience might seem surprising at first sight but it is conceivable that some psychological factors, such as anxiety or optimism, affect both perceived risk and risk-taking, while the same psychological factors do not affect patience. For instance, anxiety or optimism might affect how much an individual seeks official information on the prevalence of COVID-19 cases and thus increase how high that individual perceives her morbidity and mortality risk. Concomitantly, anxiety or optimism might affect an individual's actual risk-taking by influencing that individual's perception of the costs and benefits of taking risks [56,57]. In the case of patience, however, anxiety and optimism might be more orthogonal to people's willingness to wait for rewards.

**Figure 6.**
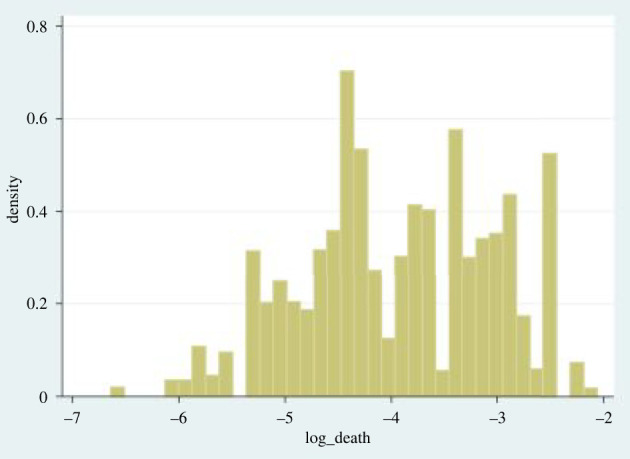
Histogram of the log of the percentage of deaths by area for each participant (*N* = 5000).

There are several limitations to our results. First, the measure of perceived risk was capped at 25%, thereby creating a potential ceiling effect. Indeed, another study on perceived risk associated with COVID-19 in the UK found that people on average perceived a 25.89% chance of being infected by COVID-19, which suggests that perceived risk may be underestimated in our study [58]. This would increase the chance of a false negative, which could explain the absence of a clear effect of perceived risk on patience. Finally, our measure of patience may also fail to capture the full range of preferences. From the distribution of patience, we can see that many individuals reported the lowest level of patience (27.44%, see appendix, figure 6). Again, this would increase the chance of a false negative, so that further investigation of the effect of perceived morbidity and mortality risk is warranted.

## 5. Conclusion

In the present research, we were able to show that people form their expectations about morbidity and mortality risk in a rational way by using the available information in their environment. The first months of the pandemic in France were characterized by a lot of country-level information about the prevalence of the virus and a desire from the government to raise people's awareness about the risks associated with COVID-19. The fact that people were able to take into account very local information about the risk they were personally exposed to shows that, even in high-stress environments, people form rational expectations. We also identified a causal effect of perceived morbidity and mortality risk on risk-taking. This shows that people adapt their behaviour to new circumstances in their environment, including potential future adversity in the form of a health shock.

Is the effect of perceived morbidity and mortality risk durable? Our study took place after the first confinement in France, in June 2020. Since then, the French population has been exposed to many more months of the pandemic and social distancing measures. It is possible that people have completely adapted to their new environment such that perceived morbidity and mortality risk no longer has an effect on their risk-taking and patience. In addition, what will happen once the pandemic is over and the morbidity and mortality risks have gone back to the prior level, remains an open question. Will people's risk-taking revert to pre-pandemic levels, or will they be durably changed? Given the central importance of risk preferences in decision-making, the potential long-term psychological effects of the COVID-19 pandemic should be addressed in future large-scale studies.

## Figures and Tables

**Figure 4.**
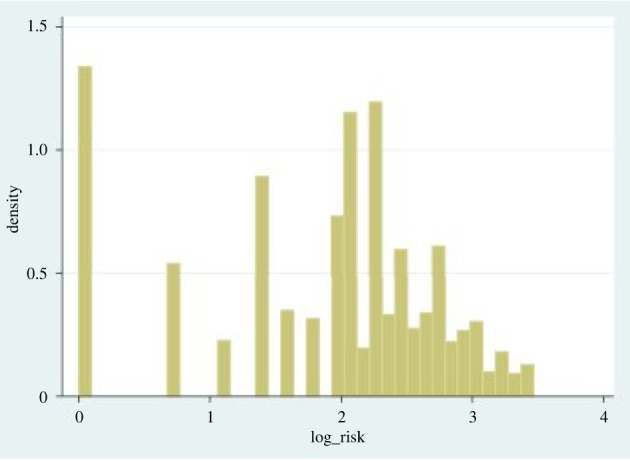
Histogram of the log of risk-taking (*N* = 5000).

**Figure 5.**
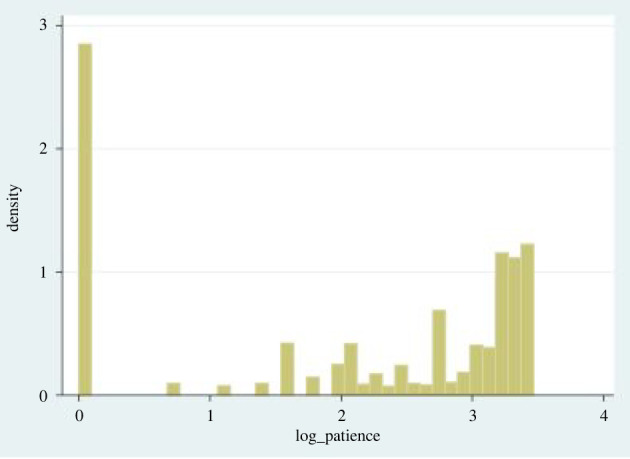
Histogram of the log of patience (*N* = 5000).

**Figure 7.**
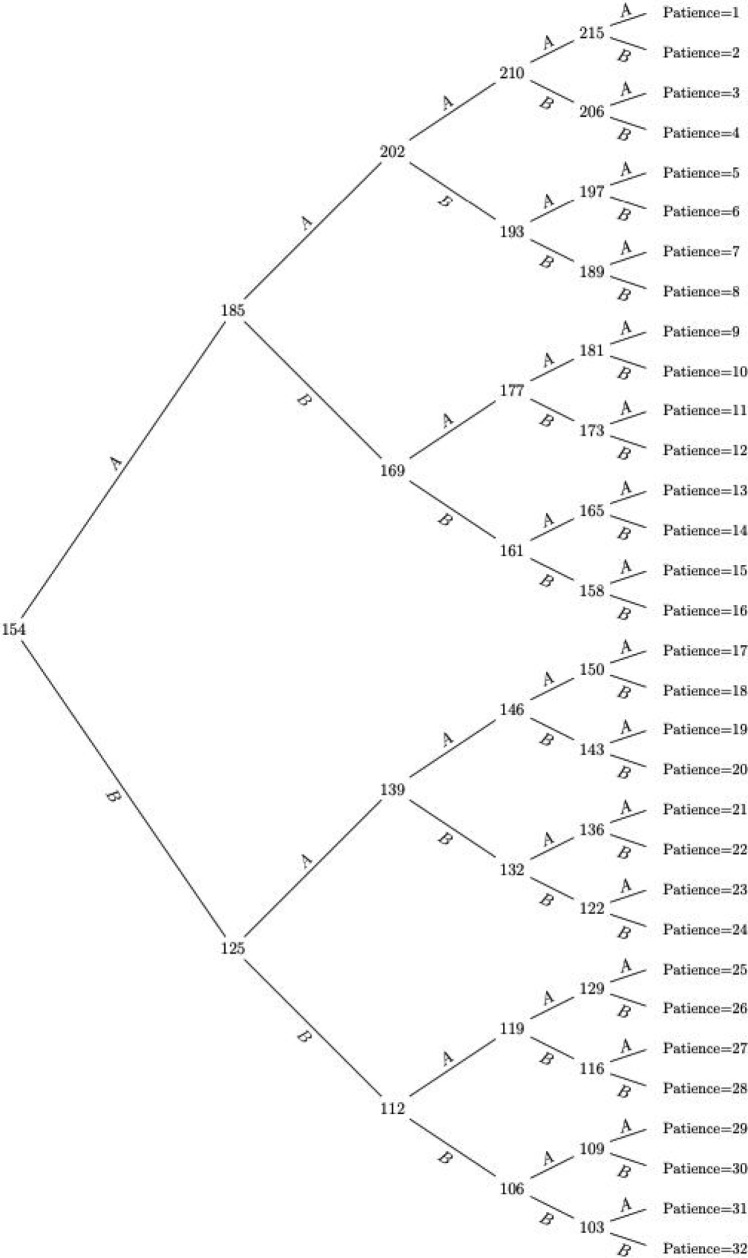
Staircase method for time discounting. Tree for the staircase time task (numbers = payment in 12 months, A = choice of ‘100€ today’, B = choice of ‘x€ in 12 months'). This figure is reproduced from Falk *et al*. [50]. Notes. The staircase procedure worked as follows. First, each respondent was asked whether they would prefer to receive 100€ today or 154€ in 12 months from now (leftmost decision node). In the case when the respondent opted for the payment today (A), in the second question the payment in 12 months was adjusted upwards to 185€. If, on the other hand, the respondent chose the payment in 12 months, the corresponding payment was adjusted down to 125€. Working further through the tree follows the same logic.

**Figure 8.**
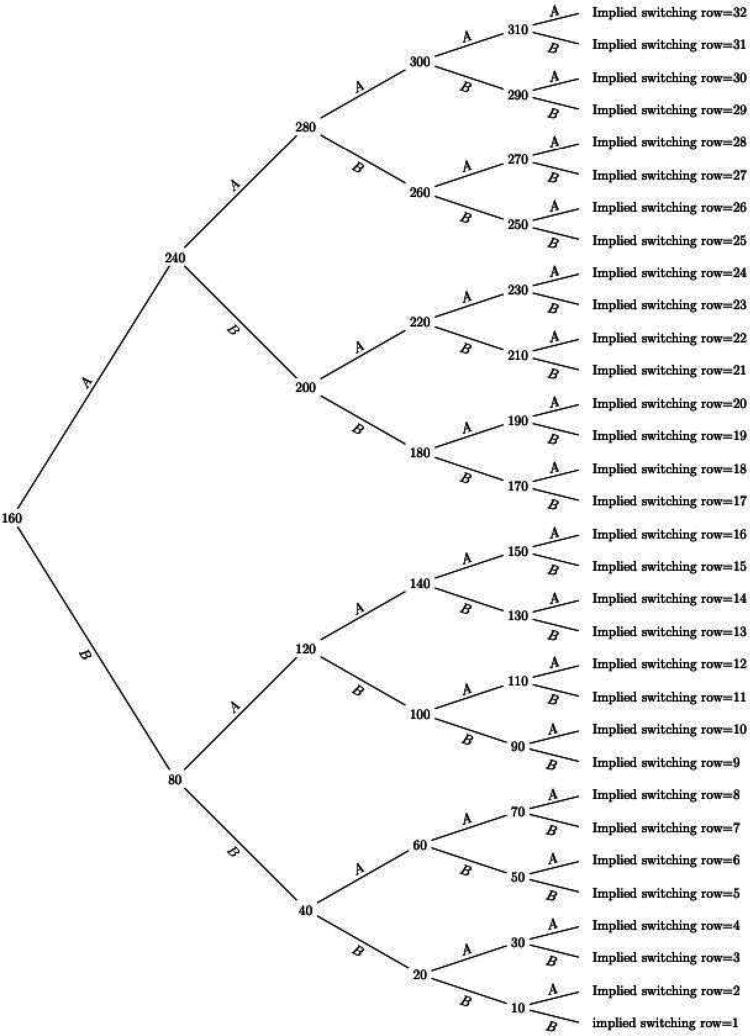
Staircase method for risk aversion. Tree for the staircase risk task (numbers = sure payment, A = choice of sure payment, B = choice of lottery). This figure is reproduced from Falk *et al*. [50]. Notes. The staircase procedure worked as follows. First, each respondent was asked whether they would prefer to receive 160€ for sure or whether they preferred a 50 : 50 chance of receiving 300€ or nothing. In case the respondent opted for the safe choice (B), the safe amount of money being offered in the second question decreased to 80€. If, on the other hand, the respondent opted for the gamble (A), the safe amount was increased to 240€. Working further through the tree follows the same logic.

## Data Availability

The full database associated with the research, along with the code, are available to the public at the following address: https://osf.io/k9uay/?view_only=eba1fcf4fb2541b3b47281c9aa7e1912. In addition, official statistics are available on the government website: https://www.data.gouv.fr/fr/datasets/donnees-hospitalieres-relatives-a-lepidemie-de-COVID-19/ and the raw data collected by the DataCovid citizen project can be downloaded on their website: https://datacovid.org/data/ under ‘Vague 8’. The STATA scripts to reproduce the analyses presented in the current research will be made available to the public at the following address: https://osf.io/k9uay/.

## Footnotes

1 The General Data Protection Regulation (GDPR) is a regulation in EU law on data protection and privacy in the European Union (EU) aimed primarily at giving people control over their personal data.

## Appendix A

**A.1. Data description**

See figures 3–8.

**A.2. Data analysis**

See tables 6–9.

**Table 6.** This table shows an ordered probit model for perceived risk as a function of the log of percentage deaths in the individual's area, whether that person worked outside their home during the first confinement, whether that person was a health worker, and a number of demographic variables. The age group is 35–44 years old, the reference income group is 1000–1500 euros per month, and the reference group for education is completed high school. *N* = 3353. We excluded participants who responded ‘I don't know’ to one or more questions about perceived risk and respondents who responded ‘I prefer not to say’ for the question about their income.

predictor variable	coefficient	s.e.
percentage death	0.134***	0.019
work outside their home	0.316***	0.051
health worker	0.806***	0.227
sex (female)	0.083*	0.041
age 18–24	0.068	0.080
age 25–34	0.013	0.080
age 45–54	–0.192**	0.072
age 55–64	–0.304***	0.072
age 65 and over	–0.122+	0.067
income (less than 500 euros)	–0.121	0.128
income (500–800 euros)	–0.110	0.112
income (800–1000 euros)	–0.037	0.113
income (1500–2000 euros)	0.053	0.078
income (2000–3000 euros)	0.097	0.066
income (more than 3000 euros)	0.096	0.060
education: no diploma	0.011	
education: middle school	0.103	
education: training degree	–0.052	
education: 1 or 2 years of graduate study	0.028	
education: 3 year or more graduate study	0.089	
education: other	0.389	

* *p* < 0.05, ** *p* < 0.01, *** *p* < 0.001.

**Table 7.** We ran a linear regression model of risk-taking and patience on the perceived risk of losing one's job and a number of demographic variables (gender, age group, income). We found that people who perceived a higher risk of losing one's job or going bankrupt were less risk-taking (although not significantly so) and less patient.

predictor variable	risk-taking		patience	
	*b*	s.e.	*b*	s.e.
risk of losing job	–0.001	0.001	–0.004***	0.001
sex (female)	–0.133**	0.044	–0.152*	0.067
age 18–24	0.156	0.098	0.124	0.150
age 25–34	0.114**	0.042	0.143*	0.066
age 45–54	–0.011	0.045	–0.013	0.071
age 55–64	–0.069	0.065	0.006	0.086
age 65 and over	–0.190	0.152	0.207	0.193
income (less than 500 euros)	–0.534*	0.253	0.041	0.317
income (500–800 euros)	–0.166	0.199	–0.035	0.208
income (800–1000 euros)	–0.102	0.187	0.072	0.242
income (1500–2000 euros)	0.135	0.092	0.309**	0.110
income (2000–3000 euros)	0.145	0.086	0.183	0.103
income (more than 3000 euros)	0.260**	0.082	0.476***	0.092

* *p* < 0.05, ** *p* < 0.01, *** *p* < 0.001.

**Table 8.** Structural model for risk-taking and patience on perceived risk. We run the structural model to evaluate the impact of perceived risk on risk-taking and patience. To ensure the robustness of our results, we change the specification for perceived risk. We define a categorical variable with three values: low perceived risk (1% and 5%), medium perceived risk (between 5% and 20%) and high perceived risk (between 20% and 25%). We find again a negative effect of perceived morbidity and mortality risk on risk-taking.

predictor variable	risk-taking		patience	
	*b*	s.e.	*b*	s.e.
equation (2.2)				
perceived risk	–0.844***	0.074	0.060	0.289
sex (female)	–0.085**	0.033	–0.163**	0.059
age 18–24	0.270**	0.097	0.241	0.127
age 25–34	0.211**	0.069	0.198**	0.075
age 45–54	–0.088	0.073	0.000	0.072
age 55–64	–0.276***	0.074	0.014	0.101
age 65 and over	–0.248***	0.060	–0.081	0.088
income (less than 500 euros)	–0.194	0.171	–0.228	0.191
income (500–800 euros)	–0.176	0.131	–0.093	0.156
income (800–1000 euros)	–0.114	0.127	–0.128	0.167
income (1500–2000 euros)	0.185*	0.076	0.151	0.107
income (2000–3000 euros)	0.179*	0.076	0.289**	0.091
income (more than 3000 euros)	0.354***	0.074	0.532***	0.086
equation (2.1)				
perceived risk	0.095***	0.018	0.139***	0.023
sex (female)	0.052	0.036	0.060	0.038
age 18–24	–0.017	0.089	–0.056	0.088
age 25–34	0.124	0.079	0.106	0.079
age 45–54	–0.107	0.072	–0.120+	0.071
age 55–64	–0.305***	0.078	–0.323***	0.080
age 65 and over	–0.202**	0.065	–0.228***	0.067
income (less than 500 euros)	–0.156	0.140	–0.133	0.132
income (500–800 euros)	–0.077	0.127	–0.072	0.126
income (800–1000 euros)	–0.157	0.119	–0.146	0.119
income (1000–1500 euros)	0.145	0.086	0.158	0.085
income (2000–3000 euros)	0.097	0.068	0.099	0.068
income (more than 3000 euros)	0.107	0.067	0.111	0.068
*ρ*	0.735***	0.035	–0.031	0.212

* *p* < 0.05, ** *p* < 0.01, *** *p* < 0.001.

**Table 9.** Structural model for risk-taking and patience on perceived risk. We run the structural model to evaluate the impact of perceived risk on risk-taking and patience. To ensure the robustness of our results, we change our instrument. Instead of using the percentage of deaths in an area, we use the percentage of hospitalization. We find similar results using this new instrument.

predictor variable	risk-taking		patience	
	*b*	s.e.	*b*	s.e.
equation (2.2)				
perceived risk	–0.844***	0.074	–0.032	0.440
sex (female)	–0.085**	0.033	–0.159**	0.060
age 18–24	0.270**	0.097	0.237	0.130
age 25–34	0.211**	0.069	0.206*	0.081
age 45–54	–0.088	0.073	–0.008	0.077
age 55–64	–0.276***	0.074	–0.007	0.127
age 65 and over	–0.248***	0.060	–0.097	0.106
income (less than 500 euros)	–0.194	0.171	–0.235	0.192
income (500–800 euros)	–0.176	0.131	–0.096	0.155
income (800–1000 euros)	–0.114	0.127	–0.138	0.171
income (1500–2000 euros)	0.185*	0.076	0.162	0.116
income (2000–3000 euros)	0.179*	0.076	0.295**	0.095
income (more than 3000 euros)	0.354***	0.074	0.540***	0.093
equation (2.1)				
perceived risk	0.107***	0.019	0.158***	0.025
sex (female)	0.052	0.036	0.059	0.038
age 18–24	–0.016	0.089	–0.053	0.087
age 25–34	0.123	0.079	0.106	0.079
age 45–54	–0.107	0.072	–0.119+	0.071
age 55–64	–0.303***	0.078	–0.321***	0.080
age 65 and over	–0.205**	0.065	–0.230***	0.067
income (less than 500 euros)	–0.160	0.140	–0.141	0.135
income (500–800 euros)	–0.079	0.127	–0.076	0.126
income (800–1000 euros)	–0.157	0.119	–0.148	0.119
income (1000–1500 euros)	0.146	0.086	0.160	0.086
income (2000–3000 euros)	0.097	0.068	0.098	0.068
income (more than 3000 euros)	0.108	0.067	0.112	0.068
*ρ*	0.735***	0.035	0.036	0.322

* *p* < 0.05, ** *p* < 0.01, *** *p* < 0.001.

**A.3. Materials**

See tables 10 and 11.

**Table 10.** Measure of time discounting. Each participant was asked to answer a series of five questions from a list of 31 hypothetical choices between an early payment ‘today’ (100€) and a varying delayed payment ‘in 12 months' (starting at 103€ and increasing to 215€ in increments of 3–4€). To determine the alternatives given to an individual, we implemented a ‘staircase’ procedure. Depending on the individual's answer (payment today or in 12 months), she was given a higher or lower option in 12 months in order to find the smallest amount in 12 months for which the participant preferred the amount in 12 months to the amount today.

number	question	answer and next question
Q.2.1	please consider the following: would you rather receive 100 Euro today or 154 Euro in 12 months?	= today → go to question 2.17
		= in 12 months → go to question 2.2
Q.2.2	would you rather receive 100 Euro today or 125 Euro in 12 months?	= today → go to question 2.10
		= in 12 months → go to question 2.3
Q.2.3	would you rather receive 100 Euro today or 112 Euro in 12 months?	= today → go to question 2.7
		= in 12 months → go to question 2.4
Q.2.4	would you rather receive 100 Euro today or 106 Euro in 12 months?	= today → go to question 2.6
		= in 12 months → go to question 2.5
Q.2.5	would you rather receive 100 Euro today or 103 Euro in 12 months?	= today [final question]
		= in 12 months [final question]
Q.2.6	would you rather receive 100 Euro today or 109 Euro in 12 months?	= today [final question]
		= in 12 months [final question]
Q.2.7	would you rather receive 100 Euro today or 119 Euro in 12 months?	= today → go to question 2.8
		= in 12 months → go to question 2.9
Q.2.8	would you rather receive 100 Euro today or 122 Euro in 12 months?	= today [final question]
		= in 12 months [final question]
Q.2.9	would you rather receive 100 Euro today or 116 Euro in 12 months?	= today [final question]
		= in 12 months [final question]
Q.2.10	would you rather receive 100 Euro today or 139 Euro in 12 months?	= today → go to question 2.14
		= in 12 months → go to question 2.11
Q.2.11	would you rather receive 100 Euro today or 132 Euro in 12 months?	= today → go to question 2.13
		= in 12 months → go to question 2.12
Q.2.12	would you rather receive 100 Euro today or 129 Euro in 12 months?	= today [final question]
		= in 12 months [final question]
Q.2.13	would you rather receive 100 Euro today or 136 Euro in 12 months?	= today [final question]
		= in 12 months [final question]
Q.2.14	would you rather receive 100 Euro today or 146 Euro in 12 months?	= today → go to question 2.16
		= in 12 months → go to question 2.15
Q.2.15	would you rather receive 100 Euro today or 143 Euro in 12 months?	= today [final question]
		= in 12 months [final question]
Q.2.16	would you rather receive 100 Euro today or 150 Euro in 12 months?	= today [final question]
		= in 12 months [final question]
Q.2.17	would you rather receive 100 Euro today or 185 Euro in 12 months?	= today → go to question 2.18
		= in 12 months → go to question 2.25
Q.2.18	would you rather receive 100 Euro today or 202 Euro in 12 months?	= today → go to question 2.22
		= in 12 months → go to question 2.19
Q.2.19	would you rather receive 100 Euro today or 193 Euro in 12 months?	= today → go to question 2.20
		= in 12 months → go to question 2.21
Q.2.20	would you rather receive 100 Euro today or 197 Euro in 12 months?	= today [final question]
		= in 12 months [final question]
Q.2.21	would you rather receive 100 Euro today or 189 Euro in 12 months?	= today [final question]
		= in 12 months [final question]
Q.2.22	would you rather receive 100 Euro today or 210 Euro in 12 months?	= today → go to question 2.23
		= in 12 months → go to question 2.24
Q.2.23	would you rather receive 100 Euro today or 215 Euro in 12 months?	= today [final question]
		= in 12 months [final question]
Q.2.24	would you rather receive 100 Euro today or 206 Euro in 12 months?	= today [final question]
		= in 12 months [final question]
Q.2.25	would you rather receive 100 Euro today or 169 Euro in 12 months?	= today → go to question 2.29
		= in 12 months → go to question 2.26
Q.2.26	would you rather receive 100 Euro today or 161 Euro in 12 months?	= today → go to question 2.28
		= in 12 months → go to question 2.27
Q.2.27	would you rather receive 100 Euro today or 158 Euro in 12 months?	= today [final question]
		= in 12 months [final question]
Q.2.28	would you rather receive 100 Euro today or 165 Euro in 12 months?	= today [final question]
		= in 12 months [final question]
Q.2.29	would you rather receive 100 Euro today or 177 Euro in 12 months?	= today → go to question 2.31
		= in 12 months → go to question 2.30
Q.2.30	would you rather receive 100 Euro today or 173 Euro in 12 months?	= today [final question]
		= in 12 months [final question]
Q.2.31	would you rather receive 100 Euro today or 181 Euro in 12 months?	= today [final question]
		= in 12 months [final question]

**Table 11.** Measure of risk aversion. Each participant was asked to answer a series of five questions from a list of 31 hypothetical choices between a lottery (300€ with a 50% chance and 0€ with a 50% chance) and varying safe options (starting at 0€ and increasing to 300€ in increments of 10€). To determine the alternatives given to an individual, we implemented a ‘staircase’ procedure. Depending on the individual's answer (the sure payment or the lottery), she was a higher or lower safe option in order to find the smallest safe amount for which the participant preferred the safe amount to the lottery.

number	question	answer and next question
Q.1.1	What would you prefer: a draw with a 50 per cent chance of receiving 300 Euro, and the same 50 per cent chance of receiving nothing, or the amount of 160 Euro as a sure payment?	= 50/50 chance → go to question 1.17
		= sure payment → go to question 1.2
Q.1.2	Would you prefer the 50/50 chance or the amount of 80 Euro as a sure payment?	= 50/50 chance → go to question 1.10
		= sure payment → go to question 1.3
Q.1.3	Would you prefer the 50/50 chance or the amount of 40 Euro as a sure payment?	= 50/50 chance → go to question 1.4
		= sure payment → go to question 1.7
Q.1.4	Would you prefer the 50/50 chance or the amount of 60 Euro as a sure payment?	= 50/50 chance → go to question 1.5
		= sure payment → go to question 1.6
Q.1.5	Would you prefer the 50/50 chance or the amount of 70 Euro as a sure payment?	= 50/50 chance → go to next section
		= sure payment → go to next section
Q.1.6	Would you prefer the 50/50 chance or the amount of 50 Euro as a sure payment?	= 50/50 chance → go to next section
		= sure payment → go to next section
Q.1.7	Would you prefer the 50/50 chance or the amount of 20 Euro as a sure payment?	= 50/50 chance → go to question 1.8
		= sure payment → go to question 1.9
Q.1.8	Would you prefer the 50/50 chance or the amount of 30 Euro as a sure payment?	= 50/50 chance → go to next section
		= sure payment → go to next section
Q.1.9	Would you prefer the 50/50 chance or the amount of 10 Euro as a sure payment?	= 50/50 chance → go to next section
		= sure payment → go to next section
Q.1.10	Would you prefer the 50/50 chance or the amount of 120 Euro as a sure payment?	= 50/50 chance → go to question 1.14
		= sure payment → go to question 1.11
Q.1.11	Would you prefer the 50/50 chance or the amount of 100 Euro as a sure payment?	= 50/50 chance → go to question 1.13
		= sure payment → go to question 1.12
Q.1.12	Would you prefer the 50/50 chance or the amount of 90 Euro as a sure payment?	= 50/50 chance → go to next section
		= sure payment → go to next section
Q.1.13	Would you prefer the 50/50 chance or the amount of 110 Euro as a sure payment?	= 50/50 chance → go to next section
		= sure payment → go to next section
Q.1.14	Would you prefer the 50/50 chance or the amount of 140 Euro as a sure payment?	= 50/50 chance → go to question 1.15
		= sure payment → go to question 1.16
Q.1.15	Would you prefer the 50/50 chance or the amount of 150 Euro as a sure payment?	= 50/50 chance → go to next section
		= sure payment → go to next section
Q.1.16	Would you prefer the 50/50 chance or the amount of 130 Euro as a sure payment?	= 50/50 chance → go to next section
		= sure payment → go to next section
Q.1.17	Would you prefer the 50/50 chance or the amount of 240 Euro as a sure payment?	= 50/50 chance → go to question 1.25
		= sure payment → go to question 1.18
Q.1.18	Would you prefer the 50/50 chance or the amount of 200 Euro as a sure payment?	= 50/50 chance → go to question 1.22
		= sure payment → go to question 1.19
Q.1.19	Would you prefer the 50/50 chance or the amount of 180 Euro as a sure payment?	= 50/50 chance → go to question 1.20
		= sure payment → go to question 1.21
Q.1.20	Would you prefer the 50/50 chance or the amount of 190 Euro as a sure payment?	= 50/50 chance → go to next section
		= sure payment → go to next section
Q.1.21	Would you prefer the 50/50 chance or the amount of 170 Euro as a sure payment?	= 50/50 chance → go to next section
		= sure payment → go to next section
Q.1.22	Would you prefer the 50/50 chance or the amount of 220 Euro as a sure payment?	= 50/50 chance → go to question 1.23
		= sure payment → go to question 1.24
Q.1.23	Would you prefer the 50/50 chance or the amount of 230 Euro as a sure payment?	= 50/50 chance → go to next section
		= sure payment → go to next section
Q.1.24	Would you prefer the 50/50 chance or the amount of 210 Euro as a sure payment?	= 50/50 chance → go to next section
		= sure payment → go to next section
Q.1.25	Would you prefer the 50/50 chance or the amount of 280 Euro as a sure payment?	= 50/50 chance → go to question 1.29
		= sure payment → go to question 1.26
Q.1.26	Would you prefer the 50/50 chance or the amount of 260 Euro as a sure payment?	= 50/50 chance → go to question 1.27
		= sure payment → go to question 1.28
Q.1.27	Would you prefer the 50/50 chance or the amount of 270 Euro as a sure payment?	= 50/50 chance → go to next section
		= sure payment → go to next section
Q.1.28	Would you prefer the 50/50 chance or the amount of 250 Euro as a sure payment?	= 50/50 chance → go to next section
		= sure payment → go to next section
Q.1.29	Would you prefer the 50/50 chance or the amount of 300 Euro as a sure payment?	= 50/50 chance → go to question 1.31
		= sure payment → go to question 1.30
Q.1.30	Would you prefer the 50/50 chance or the amount of 290 Euro as a sure payment?	= 50/50 chance → go to next section
		= sure payment → go to next section
Q.1.31	Would you prefer the 50/50 chance or the amount of 310 Euro as a sure payment?	= 50/50 chance → go to next section
		= sure payment → go to next section
